# Kinetic degradation of reactive dye RED 243 using potassium peroxymonosulfate with zero-valent iron particles

**DOI:** 10.1007/s11356-026-37856-z

**Published:** 2026-05-29

**Authors:** Lucas Alcides Moreira Santos, Eduardo Soares de Alcântara Queiroz, Ricardo Gabbay de Souza, Lídia Yokoyama, Felipe Sombra dos Santos

**Affiliations:** 1 Federal University of Rio de Janeiro Chemistry School, Universidade Federal do Rio de Janeiro–UFRJ, Rio de Janeiro, Brazil; 2 Technical University of Denmark, Danmarks Tekniske Universitet–DTU, Lyngby Campus, Lyngby, Denmark

**Keywords:** Reactive dye, Zero valent iron, Advanced oxidation process, PMS, Reactional kinetic, Dye degradation

## Abstract

The degradation of persistent organic pollutants reduces their harmful effects on the environment. Various treatment techniques are employed to remediate these pollutants, and among them, advanced oxidative processes are effective for breaking down recalcitrant compounds. This manuscript investigated the degradation of the dye RED 243 using PMS in combination with zero-valent iron particles of different sizes. The reduction in the dye’s colorimetric intensity occurred rapidly, within a few seconds. The results indicated that the reduction in colorimetric intensity ranged from 62 to 83% by the end of the tests when PMS was used with ZVI at various temperatures. It examined several types of acids to assess the impact of anions on the degradation process and found that organic acids were more efficient. The optimal reaction conditions, which minimized reagent usage, occurred at the original sample pH, 313 K, 25 mg L^−1^ PMS, and 100 mg L^−1^ micrometric zero-valent iron. Under these conditions, we achieved a degradation efficiency of 83% in colorimetric intensity. The dye degradation reaction followed pseudo-first-order kinetics with respect to dye concentration, yielding activation energies of 39.77 kJ mol^−1^ for micro-sized iron and 55.67 kJ mol^−1^ for nano-sized iron particles.

**Graphical abstract:**

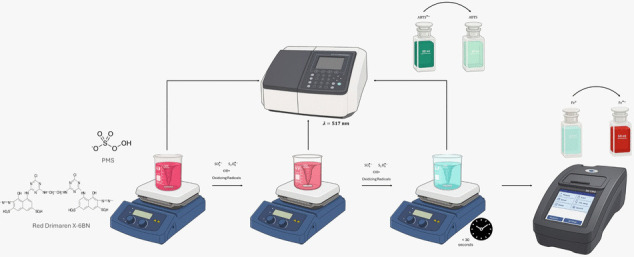

## Introduction

The complexity and diversity of textile pigments synthesized by the industry for various purposes, such as textiles, paints, papers, plastics, and tanning, corroborate a possible impact on water bodies, due to a possible discharge of solutions containing non-biodegradable dye groups in the composition of the effluent (Shokoohi et al. 2021; Zeng et al. 2018).

Conventional effluent treatment technologies present ineffective results in degrading persistent and recalcitrant pollutants. They cannot selectively remove such small quantities of these contaminants, with short reaction times and high removal efficiency (Eckenfelder 2000; Metcalf and Eddy 2014).

Advanced oxidative processes present different radical-generating species, which can be used in chemical reactions, such as hydroxyls (*E* = 1.9 to 2.7 V), sulfates (*E* = 2.5 to 3.1 V), and singlet oxygen, which act as good oxidizing agents capable of mineralizing recalcitrant compounds (Shokoohi et al. 2021; Wang et al. 2014; Wu et al. 2020). Hydroxyl and sulfate radicals exhibit good operational efficiency over a wide pH range of 4.0–9.0 (Cai et al. 2020; Guo et al. 2018; Hu and Long 2016; Wang et al. 2017; Wang and Wang 2018).

The generation of sulfate radicals (SO_4_•^−^) for the oxidative process can occur through the activation of persulfates, such as peroxymonosulfate (PMS) and peroxydisulfate (PDS), with the aid of UV, ultrasound, heating, and the use of some metals (Wu et al. 2020; Cai et al. 2020; Guo et al. 2018). Compared to hydrogen peroxide, persulfates are relatively stable in the solid state, which favors their storage and transportation (Wang et al. 2014).

Peroxymonosulfate (PMS) is a white solid reagent precursor of sulfate radicals and is an environmentally harmless, relatively low-cost oxidant capable of degrading a range of organic pollutants [12]. PMS is a white, stable powder at pH values less than or equal to 6. At pH 9, it shows low stability and decomposes into SO_5_^2−^. The bonds between oxygen atoms in the PMS structure exhibit an asymmetric geometry, with a bond length of 1.453 Å. The generated reduction potential can range from 2.5 to 3.1 V. It has a solubility in water greater than 250 g L^−1^. The most commonly used reagent is Oxone, which is based on the potassium cation (Wang and Wang 2018).

In a Fenton system, the reaction system must be acidified to favor the generation of hydroxyl and sulfate radicals. Acidity favors the predominance of sulfate radicals over hydroxyl radicals in solution. When the pH increases, the concentration of sulfate radicals decreases, allowing the formation of hydroxyl radicals due to thermodynamic predominance in an alkalizing system (Chen et al. 2019).

Some authors (Wang and Wang 2018; Ren et al. 2015; Cao et al. 2019; Deng et al. 2018; Yang et al. 2022) propose that the mechanism of sulfate radical activation through the use of the PMS reagent occurs, according to Eqs. (1) to (3), and in them the ions Fe^2+^ and Co^2+^ are suggested as the best activators, with iron being the most recommended due to its lower toxicological potential in the environment. However, other forms, such as heat, UV radiation, transition metals, carbon-based catalysts, electrolysis, and alkaline pH, can also activate the generation of sulfate radicals.

1 $$\mathrm M^{\mathrm n+}+{\mathrm{HSO}}_{5}^-\rightarrow\mathrm M^{\left(\mathrm n+1\right)+}+{\mathrm{SO}_4^-}{{\bullet}}+\mathrm{HO}^-$$

2 $$\mathrm M^{\mathrm n+}+{\mathrm{HSO}}_{5^-}\rightarrow\mathrm M^{\left(\mathrm n+1\right)+}+{\mathrm{SO}_4^-}+\mathrm{HO}^\bullet$$

3 $$\mathrm M^{\left(\mathrm n+1\right)}+{\mathrm{HSO_5^-}}\rightarrow\mathrm M^{\mathrm n+}+{\mathrm{SO_5^-}{\bullet}}+\mathrm H^+$$

Other iron-based catalysts have been used to activate PMS and promote the degradation of pollutants. Tetracycline was 94.3% removed using PMS activated by W_0.5_Ag_0.5_FeO_3_ nanoparticles (Yuennan et al. 2025). In another study also using tetracycline, 86% degradation was achieved with PMS activated by a Co_0.5_Cu_0.5_Fe_2_O_4_ photocatalyst (Chen et al. 2023). Carbon@ZnCuFeS nanoparticles with PMS were used in hydrothermal photodegradation tests of 95.8% of Rhodamine B (Ashraf et al. 2025). The degradation of Congo red was studied using PMS activated by an Ag@ZnFe2O4 nanocomposite, yielding 98.8% efficiency (Yang et al. 2024). p-Phenol sulfonic acid was 88% degraded by PMS activated with a heterogeneous CoFe_2-x_Mo_x_O_4_ catalyst (Hassam et al., 2023). The acid red (AR14) was 93.9% removed by PMS activated nZVI (Samarghandi et al. 2020). The amaranth dye was 100% degraded by PMS activated by CoFe_2_O_4_NPs (Lin et al. 2018). Azorubine was 95% removed from the system by PMS-activated FeMWCNT (Madihi-Bidgoli et al. 2021). The PMS was also activated with FeCoP@NF to separate RhB from water with 62.09% of removal (Pan et al. 2024).

The mechanism of generation of sulfate and hydroxyl radicals in the presence of zero-valent iron nanoparticles and PMS is described by reactions (4) to (12) (Cai et al. 2020; Wang et al. 2017; Cao et al. 2019; Chen et al. 2019; Deng et al. 2018; Gu et al. 2018; Santos et al. 2017, 2016).

4 $$\mathrm{Fe}^\circ+2\mathrm{Fe}^{3+}\rightarrow3\mathrm{Fe}^{2+}$$

5 $$\mathrm{Fe}^{2+}+{\mathrm{SO_4^-}}{\bullet}\rightarrow\mathrm{Fe}^{3+}+{\mathrm{SO_4^{2-}}}$$

6 $$\mathrm{Fe}^{2+}+\mathrm{HO}^\bullet\rightarrow\mathrm{Fe}^{3+}+\mathrm{OH}^-$$

7 $$\mathrm{HO}^\bullet+{\mathrm{HSO_5^-}}\rightarrow{\mathrm{SO_5^-}}+{\mathrm H}_2\mathrm O$$

8 $${\mathrm{SO_5^-}{\bullet}}+{\mathrm{SO_5^-}}{\bullet-}\rightarrow{\mathrm{SO_4^-}}{\bullet}+{\mathrm O}_2+{\mathrm{SO_4^-}}{\bullet}$$

9 $${\mathrm{SO_5^-}}{\bullet}+{\mathrm{SO_5^-}}{\bullet}\rightarrow{\mathrm S}_2{\mathrm O_8^{2-}}+{\mathrm O}_2$$

10 $$2{\mathrm{SO_5^-}}{\bullet}+2\mathrm{OH}^-\rightarrow2\mathrm{HO}\bullet+2{\mathrm{SO_4^{2-}}}+{\mathrm O}_2$$

11 $${\mathrm{SO_5^-}}{\bullet}+2{\mathrm H}_2\mathrm O\rightarrow3\;\mathrm{HO}\bullet+{\mathrm{SO_4^{2-}}}+\mathrm H^+$$

12 $${\mathrm{SO_4^-}}{{\bullet}}+\mathrm{OH}^-\rightarrow{\mathrm{SO}}_{4^{2-}}+\mathrm{HO}\bullet$$

In some cases, radical scavengers such as phenol, ethanol, and tert-butyl alcohol (TBA) are used to inhibit the action of unwanted radicals. In the liquid phase, ethanol is most commonly used to capture sulfate and hydroxyl radicals, while TBA is more selective for hydroxyl radicals (Cai et al. 2020).

Kinetic degradation models for PMS-assisted dye degradation depend on the oxidizing agent concentration, reaction temperature, and system pH. The latter is strongly linked to the chemical structure of the pollutant, which will exhibit greater acidity or alkalinity in the medium. Degradation reactions exhibit first- and pseudo-first-order kinetics in most cases (Zeng et al. 2018; Lou et al 2017; Liu et al. 2021) and second-order kinetics in some cases (Shokoohi et al. 2021; Ling et al. 2010).

The degradation kinetics of different organic pollutants using PMS under varying experimental conditions mainly exhibit pseudo-first-order kinetics, allowing the calculation of the kinetic constant and activation energy, as presented in Eqs. (13) to (16) (Ashraf et al. 2025; Chen et al. 2023; Hassan et al. 2023; Yang et al. 2024; Yuennan et al. 2025).

13 $$\tt {C}_{t}={C}_{o}\cdot {e}^{-k\cdot t}$$

14 $$\mathrm{l}\mathrm{n}\left(\frac{{\mathrm{C}}_{\mathrm{t}}}{{\mathrm{C}}_{\mathrm{o}}}\right)=-\mathrm{k}\cdot \mathrm{t}$$

15 $$\mathrm{k}={\mathrm{k}}_{\mathrm{o}}\cdot {\mathrm{e}}^{\frac{-{\mathrm{E}}_{\mathrm{a}}}{\mathrm{R}\cdot \mathrm{t}}}$$

16 $$\tt ln{k}=ln{k}_{o}-\frac{{E}_{a}}{R\cdot t}$$

However, systems containing chloride ions in solution inhibit the reaction kinetics of the sulfate radical. In this case, the sulfate radical oxidizes the chloride ion to a less reactive species (the chloride radical), as shown in Eq. (17), via electron transfer (Huang et al. 2018; Wang et al. 2011).

17 $${\mathrm{S}\mathrm{O}}_{4}^{-}\bullet + {\mathrm{C}\mathrm{l}}^{-}\to {\mathrm{S}\mathrm{O}}_{4}^{2-}+{\mathrm{C}\mathrm{l}}^{\bullet }; {\mathrm{k}}_{\mathrm{f}}=\left(\mathrm{3.2}\pm \mathrm{0,2}\right)\times {10}^{8}{\mathrm{M}}^{-1}{\mathrm{s}}^{-1}$$

The degradation kinetics of Congo Red and Rhodamine B dyes using PMS and a CoFeNi catalyst showed that the reaction is first-order with respect to dye concentration, and the PMS activation rate is pseudo-first-order with respect to the HSO_5_^−^ concentration (Zeng et al. 2018).

For the degradation of the acidic dye Orange 7, it was observed that polyphosphate with PMS significantly increased the degradation rate constant with increasing oxidizing agent concentration and pH, especially at pH 7–10, adjusted with NaOH solution. The dye removal efficiency at pH 10.0 was 98.2%, occurring in 500 s (Lou et al. 2017).

During degradation tests of the reactive dye Black 5 (RB5) to evaluate reaction kinetics in the presence of PMS, a favorable reaction was observed at an acidic pH (4.0). Excess PMS at concentrations above 12.27 mM slowed the kinetics by generating sulfate radical scavengers in solution. It was observed that RB5 removal follows pseudo-first-order degradation kinetics, with PMS concentration, reaction temperature, and initial pH as independent variables that affect the model (Liu et al. 2021).

The degradation of the dye RED 243 by advanced oxidation systems has already been studied in the literature, employing different catalysts and oxidizing agents (Araujo et al. 2011; Görücü et al. 2023; Kavci 2021; Pandis et al. 2022; Santos et al. 2020). However, the experimental evaluation of the dye degradation kinetics using PMS remains valid.

Thus, this research aimed to evaluate the use of PMS to promote the degradation of a RED 243 reactive dye solution containing zero-valent iron particles under various experimental conditions, thereby obtaining kinetic parameter data.

## Materials and methods

Peroxymonosulfate (PMS) is used as a sulfate radical precursor reagent and is an environmentally friendly, relatively low-cost oxidant capable of degrading a range of organic pollutants [12]. The generation of radicals capable of promoting pollutant oxidation involves sulfate radical activation, for which the use of Fe^2+^ and Co^2+^ metal ions as precursors is suggested, with emphasis on iron due to its lower environmental toxicological potential (Deng et al. 2022).

Potassium peroxymonosulfate (Oxone®) was purchased from Sigma–Aldrich. Zero valent iron, responsible for generating Fe^2+^ ions in solution, was used at the 25 nm nanoscale, with purity above 95%, obtained from mkNANO. Micron-scale iron (purity > 99%) purchased from Sigma–Aldrich was used to investigate the performance of iron at larger particle sizes. The Drimaren X-6BN 150 reactive red dye (C.I. Reactive Red 243), RED 243, was supplied by Clariant.

Using a stock dye solution with a concentration of 20 mg∙L^−1^, the tests were performed under different reaction conditions of pH, temperature, dye concentration of 10 mg∙L^−1^, PMS concentrations (25 to 50 mg L^−1^) with micro and nanoscale elemental iron (50 to 200 mg L^−1^), and without the use of iron in solution.

The tests were performed in triplicate for 50 min, during which aliquots were removed every 5 to 10 min to monitor the degradation of the dye’s chromophore group by spectrophotometric analysis at 517 nm (Santos et al. 2016).

The degradation kinetics tests were performed in triplicate to obtain results for evaluating the kinetic parameters. Reagent concentrations were reduced while maintaining the same dye concentration (10 mg L^−1^), and aliquots were withdrawn every 1 to 2 min to monitor the degradation. PMS was used at a concentration of 6.25 mg L^−1^, micrometric iron at 25 mg L^−1^, and organic acids were quantitatively diluted in water at a 1:3 ratio.

The test to evaluate the residual PMS concentration in the solution was performed in triplicate using the ABTS colorimetric method. A 5.0 mL aliquot was collected and filtered with a 0.45-μm membrane syringe filter. Next, 5.0 mL of demineralized water, 0.4 mL of ABTS (10 mM), and 0.2 mL of a Co^2+^ solution (10 mM) should be immediately added to the sample, and the sample should be allowed to stand for 10 min for a green color to appear due to the formation of the ABTS^+^ cation. The resulting solution should be analyzed using a UV/Visible spectrophotometer at a wavelength of 735 nm (Wang et al. 2014).

To determine the residual iron concentration in solution, use the Ferrover program of the HACH DR 3900 spectrophotometer with the specific reagent for this determination, purchased from the equipment manufacturer. A 10 mL aliquot of the sample is collected, filtered with a 0.45-μm membrane syringe filter, and added to a beaker. Then, 15.0 mL of demineralized water must be added to adjust the volume and concentration of the analyte to the method’s analysis range, and wait 3 min before proceeding with the reading.

## Results and discussion

### Results of the investigation of dye degradation without the addition of iron

**Fig. 1:**
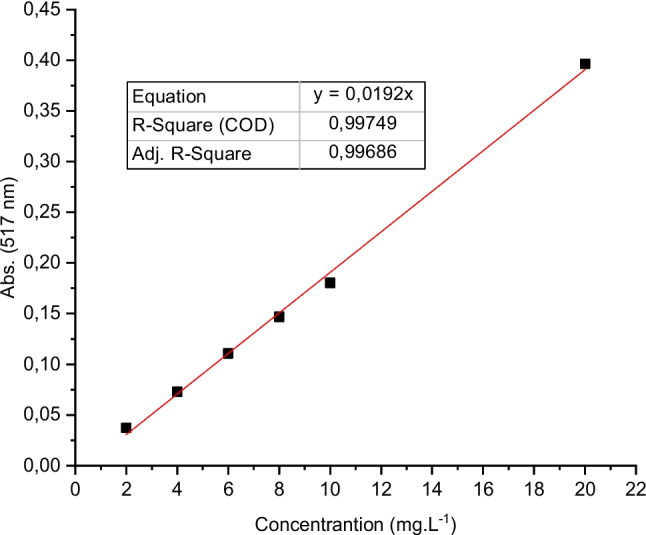
RED 243 dye calibration standard curve

The first step in investigating the color degradation of the dye solution was to determine the wavelength of the highest absorption peak, which occurred at 517 nm. The standard correlation curve for the dye solution is shown in Fig. 1.

Next, we investigated whether color degradation occurred only in tests containing PMS without adding ZVI. Room temperature was used for all tests; the PMS concentration was 50 mg L^−1^, and the pH values were 3.0, 4.0, 6.0, 7.0, and 8.0. Initial pH adjustments were made with 1.0 M HCl and 0.1 M NaOH solutions. The results presented are the averages obtained from triplicate tests.

**Fig. 2:**
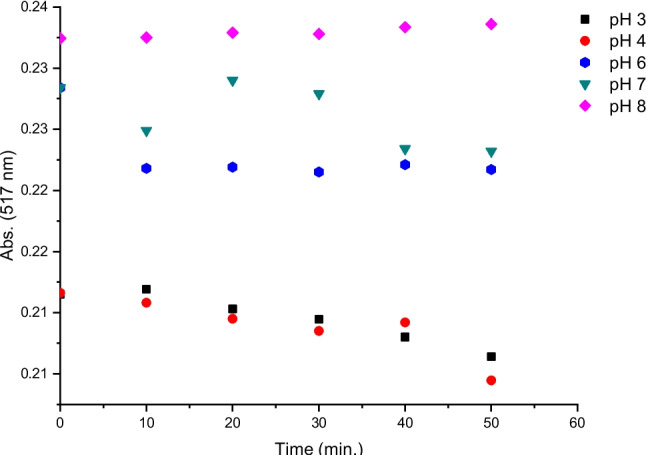
Degradation profile of the dye’s colorimetric intensity without adding Fe^0^

Since no significant color degradation was observed, the test was then performed with twice the PMS solution concentration (100 mg L^−1^), and no color degradation was observed in either case. The dye degradation at different pH values tested, without iron, is shown in Fig. 2, where the maximum degradation was 3.34%, which is practically negligible, contributing to the lack of pH variation during the reaction. It was observed that the pH practically did not vary over time.

It is believed that color degradation did not occur because sulfate and hydroxyl radical generation were not significant, as they are unfavorable to the analyzed pollutant. Therefore, pigment discoloration did not occur because there was no oxidizing agent.

It is important to emphasize that the presence of halide ions in systems containing PMS introduces dual interference in the process kinetics. This interference can be inhibitory, when the ions are in the concentration range of 0.05 to 10 mM, reducing the reaction rate, or positive, when the chloride concentration is above 50 mM, favoring the degradation kinetics (Deng et al. 2022).

The generation of active chlorine radicals explains this phenomenon via reaction with PMS. Therefore, it is essential to note that pH adjustment of the reaction medium using hydrogen halides should be performed carefully, taking this effect into account and controlling the concentration of halide ions in the assay, which may occur if demineralized water is not used to prepare the solutions.

On the other hand, organic acids did not initially exhibit any inhibitory activity against degradation; rather, they proved an efficient option for pH adjustment.

### Results of the investigation of dye degradation with iron addition

**Fig. 3:**
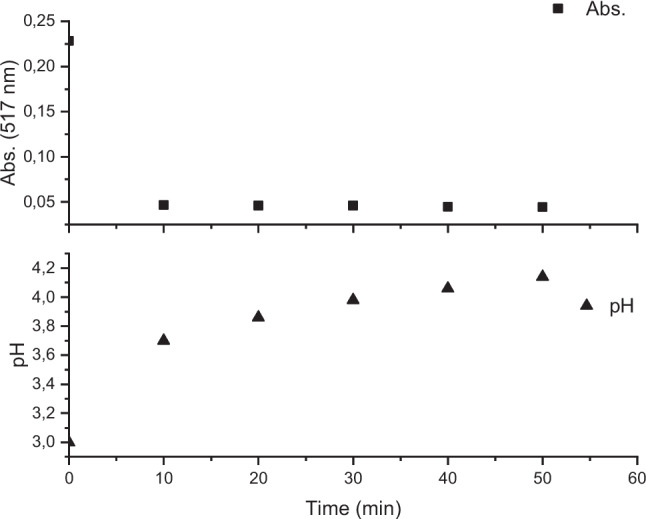
Degradation profile of RED 243 with a dye solution of 10 mg L^−1^; 50 mg L^−1^ of PMS; 200 mg L^−1^ Fe^0^, and pH equal to 3.0

The tests using elemental iron were carried out at a concentration of 200 mg L^−1^, with a solution containing 50 mg L^−1^ of PMS and 10 mg L^−1^ of the dye, at room temperature, with the pH adjusted to 3.0. The results obtained are presented in Fig. 3.

After adding the elemental iron nanoparticles, the solution was completely colorless within approximately 30 s, with no trace of the dye remaining. As the test progressed, no significant change in absorbance values was observed. After 50 min of testing, a decrease of approximately 75% was observed. A pH change of approximately one unit was also observed, reaching 4.14.

**Fig. 4:**
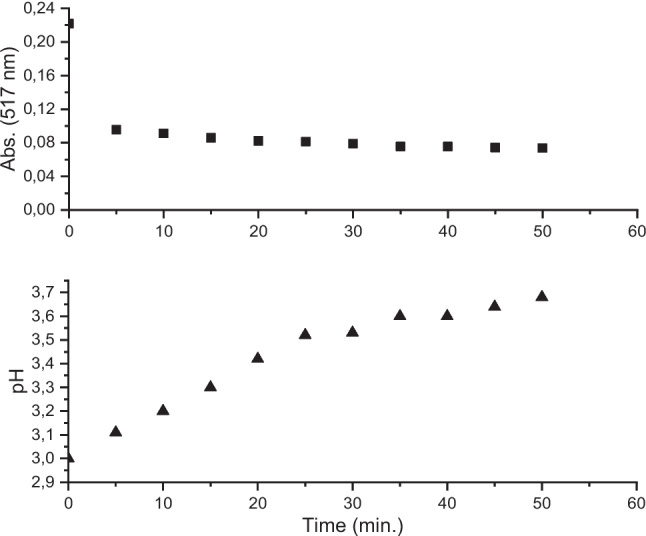
Degradation profile of RED 243 with a dye solution of 10 mg L^−1^; 25 mg L^−1^ of PMS; 100 mg L^−1^ Fe^0^, and pH equal to 3.0

To verify the impact of reducing PMS concentration on degradation performance while maintaining the experimental conditions, the PMS and iron concentrations were reduced by half (25 mg L^−1^ and 100 mg L^−1^, respectively), as shown in Fig. 4. Because the reaction occurred quickly, the time required to withdraw aliquots was reduced to verify improved behavior and degradation rate.

Comparing Figs. 3 and 4, it was observed that pH varied slightly with reduced amounts of PMS and nZVI. Color degradation was observed in approximately 15 min. At the end of the test, a 67% decline was observed, which is somewhat positive, given the reduced reagent consumption throughout the process.

**Fig. 5:**
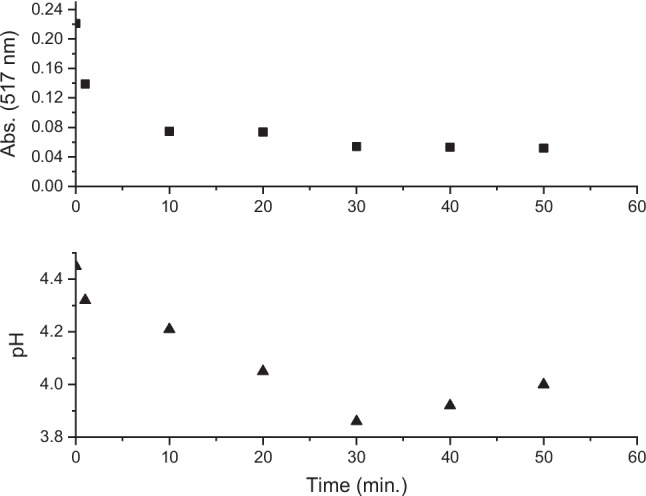
Degradation profile of RED 243 with 10 mg L^−1^ dye solution; 25 mg L^−1^ PMS; 100 mg L^−1^ Fe^0^, without pH adjustment

Another essential step was verifying that discoloration occurred without requiring a pH adjustment to the solution. The test was performed at the sample’s original pH, which ranged from 4.0 to 4.5. It was observed that solution degradation continued to occur efficiently after 30 min. The pH remained virtually unchanged over time. At the end of the test, the color removal efficiency was approximately 74%, as shown in Fig. 5. This indicates a positive outcome, as degradation is facilitated with less reagent use and a milder reaction condition.

**Fig. 6:**
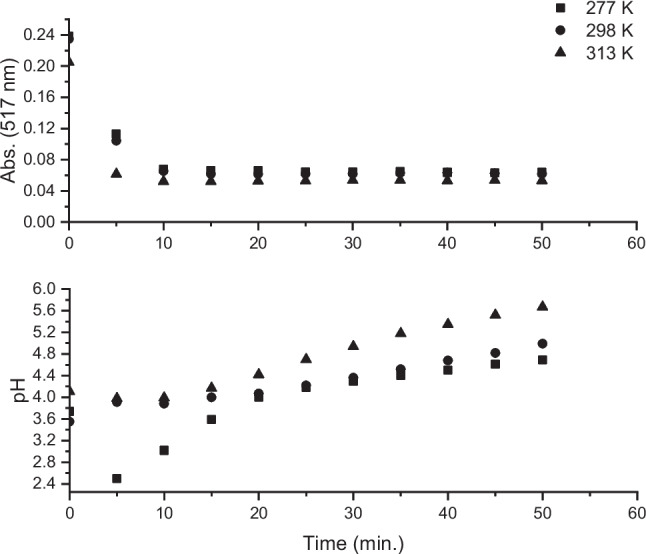
Degradation profile of RED 243 with 10 mg L^−1^ dye solution, 25 mg L^−1^ PMS, 100 mg L^−1^ Fe^0^ at original pH, at different temperatures

A test was performed with a PMS concentration of 25 mg L^−1^, micrometric elemental iron particles at 100 mg L^−1^, and the original pH at different temperatures: in an ice bath (where the temperature varied between 277 K), in a hot bath (313 K), and at room temperature (298 K), as shown in Fig. 6.

The degradation of the dye solution containing micrometric iron particles was observed to be efficient, making the process more accessible. For the dye solution, the color removal efficiency was 74%–77% in the ice bath and room-temperature systems, respectively. At 313 K, a higher color removal efficiency (83%) was observed, which differs from the other conditions, demonstrating that temperature slightly influences the process. In the textile industry, hot dyeing is common because it favors color impregnation into the fabric; often, residual dye loads reach temperatures above 313 K (Hu and Long 2016), suggesting even better degradation performance.

**Fig. 7:**
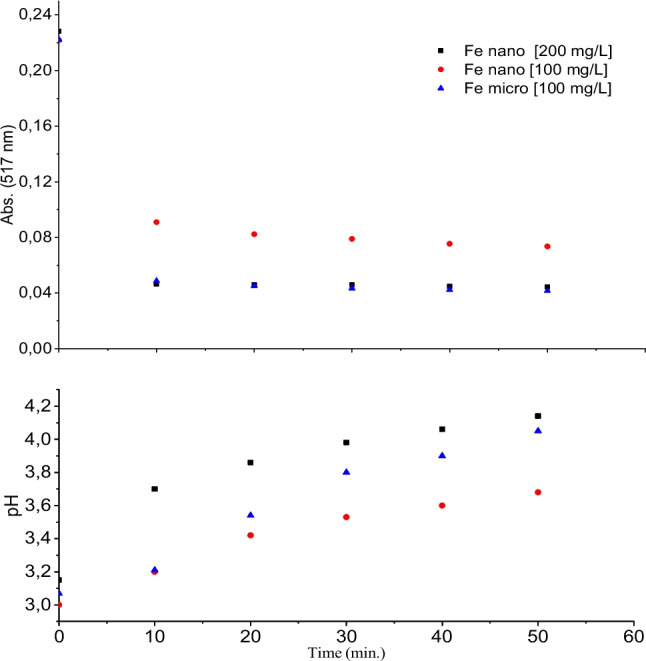
Degradation profile of RED 243 with dye solution of 10 mg L^−1^; 25 mg L^−1^ of PMS; 100 mg L^−1^ of micrometric and nanometric Fe^0^; and 50 mg L^−1^ of PMS; 200 mg L^−1^ of nanometric Fe^0^, pH equal to 3.0

The performance of iron particles at pH 3.0 was also verified against other scenarios to assess degradation (Fig. 7). The most efficient decay was observed with nanoscale elemental iron (Fe) particles at 200 mg L^−1^ and a PMS concentration of 50 mg L^−1^, in which discoloration (80% removal) occurred early in the reaction. However, in a scenario with lower reagent usage, 72% degradation was obtained using a 100 mg L^−1^ micrometric Fe^0^ solution and a PMS concentration of 25 mg L^−1^. There were variations in pH readings between 0.5 and 1, with the differences more pronounced for microscale iron.

Still on Fig. 7, it is possible to note that the color removal for nanometric iron concentration of 200 mg L^−1^ and 100 mg L^−1^ of micrometric iron were practically the same, which was not expected due to a larger reaction contact area of nano iron, as it presents a higher concentration, as well as greater oxidation kinetics (Karim et al. 2016), also having better mobility in the solution and a larger surface area, due to the particle size (Santos et al. 2016). However, the results presented can be justified by the purity of the reagents, since the micrometric iron has a purity above 99%, while the nanometric iron presents a purity above 95%.

### Evaluation of the reaction rate with organic acids

**Fig. 8:**
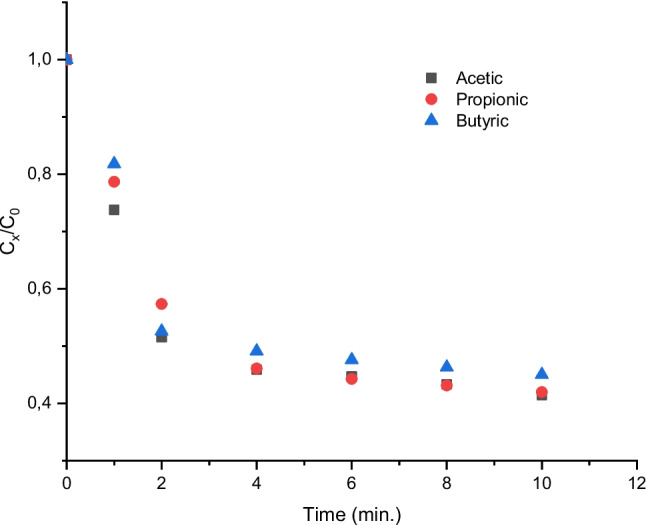
C_x_/C_0_ profile as a function of time. Degradation reaction of RED 243 with a 10 mg L^−1^ dye solution; 6.25 mg L^−1^ of PMS; 25 mg L^−1^ of micrometric Fe^0^; pH equal to 3.0

Initially, the study was conducted by performing the tests at room temperature, adjusting the pH to 3.0 with acetic acid, propionic acid, and butyric acid. Figure 8 presents the results obtained.

The use of acetic acid to adjust the pH demonstrated greater efficiency in terms of reaction speed, achieving lower concentrations in the initial minutes of the test, when the most dye molecules are available, and the rate is highest. Given the characteristics and molecular sizes of the acids used, the hypothesis explaining the observed behavior is that larger molecules result in more particles interacting with the carbon chains in the system, thereby increasing the activation energy. Among the three acids used, acetic acid had the shortest carbon chain and showed the best results. Similarly, propionic acid proved more efficient than butyric acid.

### Kinetics of dye degradation

**Fig. 9:**
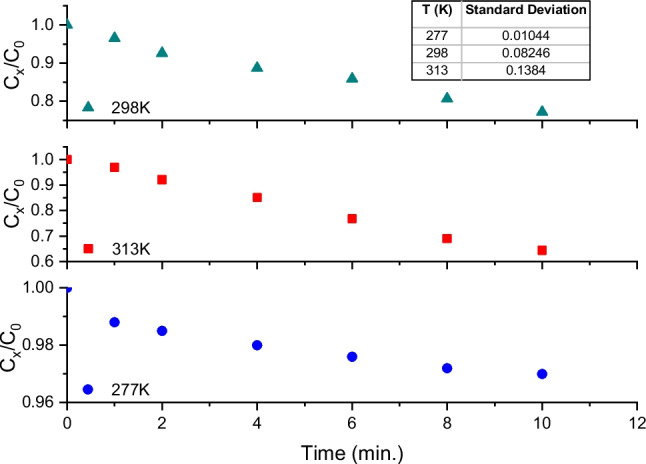
ln(C_x_)/ln(C_0_) profile as a function of time for micrometric iron at 277, 298, and 313 K temperatures

**Fig. 10:**
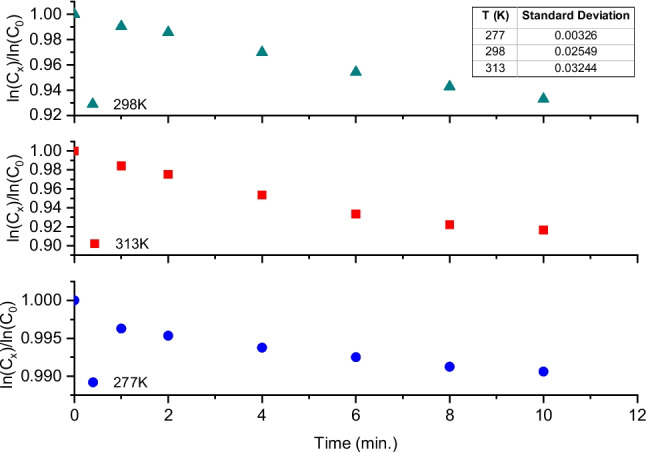
ln(C_x_)/ln(C_0_) profile as a function of time for nanometric iron at 277, 298, and 313 K temperatures

Using temperature as a parameter to study the kinetics of dye degradation, the tests were carried out at 277, 298, and 313 K. The results are presented in Fig. 9, for iron on a micrometric scale, and Fig. 10, for iron on a nanometric scale.

From these figures, it was observed that heating the system increases the reaction rate due to greater molecular agitation, as expected. Similarly, the results obtained in an ice bath demonstrated the opposite effect.

The use of micrometer-scale iron followed the trend observed in the degradation tests, achieving greater efficiency and a higher reaction rate in the initial minutes than nanometer-scale iron, due to the purity of the reagents.

Based on the concentration values obtained in the temperature effect evaluation tests, the graphical behavior of the dye degradation indicates that the reaction system obtained is pseudo-first order, with respect to the dye concentration (Amorim et al. 2009).

**Fig. 11:**
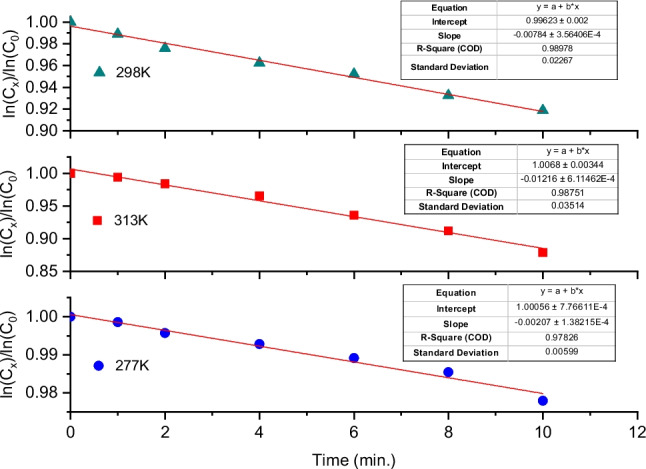
ln(C_x_)/ln(C_0_) profile as a function of time for micrometric iron at different temperatures, with the trend lines and equations of the straight lines obtained

**Fig. 12:**
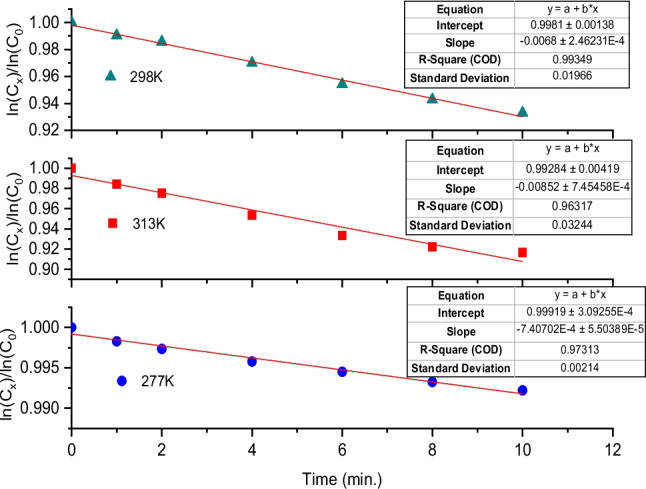
ln(C_x_)/ln(C_0_) profiles as a function of time for nanometric iron at different temperatures, with the trend lines and equations of the straight lines obtained

The results obtained are shown in Figs. 11 and 12 for micrometer- and nanometer-scale iron, respectively.

**Fig. 13:**
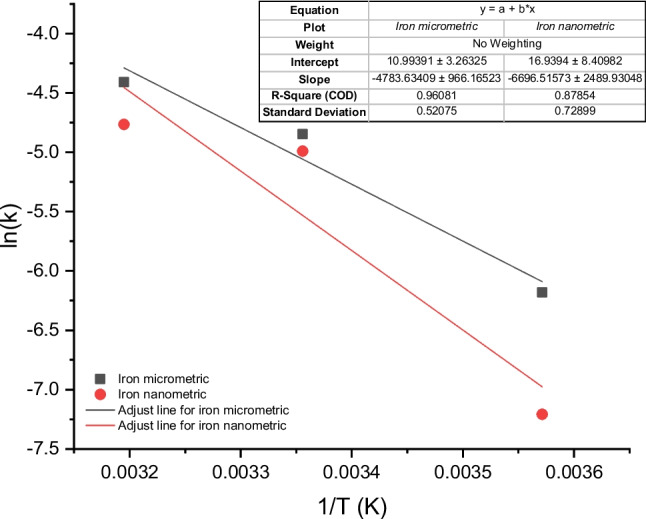
ln(k) profile as a function of the inverse of the temperature for tests with micrometric and nanometric iron

With the values of kinetic constants obtained through Figs. 11 and 12 and presented in Table 1, it was possible to determine the activation energy for each system through the Arrhenius equation, as shown in Fig. 13.

**Table 1:** Kinetic constants, standard deviation, and half-life time for different systems

T (K)	Nanometric iron			Micrometric iron		
	k (min^−1^)	σ	t_1/2_ (min)	k (min^−1^)	σ	t_1/2_ (min)
277	7.41 × 10^−4^ ± 5.50 × 10^−5^	0.00214	1.07 × 10^−3^	2.07 × 10^−3^ ± 1.38 × 10^−4^	0.00599	2.99 × 10^−3^
298	6.80 × 10^−3^ ± 2.46 × 10^−4^	0.01966	9.81 × 10^−3^	7.84 × 10^−3^ ± 3.56 × 10^−4^	0.02267	1.13 × 10^−2^
313	8.52 × 10^−4^ ± 7.45 × 10^−4^	0.03244	1.23 × 10^−3^	1.22 × 10^−2^ ± 6.11 × 10^−4^	0.03514	1.76 × 10^−2^

In the systems, the greater the slope, the greater the energy required for the reaction to occur (Amorim et al. 2009). The results in Fig. 13 allowed the determination of activation energies of 39.77 kJ mol^−1^ for micrometric iron and 55.67 kJ mol^−1^ for nanometric iron. In the other study (Araujo et al. 2011), when the same dye was degraded using H_2_O_2_ (instead of PMS) with hematite, the activation energy obtained from the system was 443.5 kJ mol^−1^, presented in Table 2, much higher than the values obtained in this study, because of the H_2_O_2_ usage instead of PMS.

**Table 2:** Activity energy for different PMS/Fe° systems

Organic pollutants	Activation systems	Oxidant agent	Activity energy (kJ.mol^−1^)	Efficient removal (%)	Ref
Drimarene red X-6BN (CI reactive red 243)	Hematite	H_2_O_2_	443.5	99.0	Araujo et al. 2011
Cibacron red FN-R (CI reactive red 238)	FeSO_4_.7H_2_O		1.09	~ 100	Núñez et al. 2007
Procion red H-E7B (CI reactive red 141)			1.26	~ 100	
Leather dye (direct red 111, DR-111)	CoSO_4_.7H_2_O	PMS	45.11	88.5	Bouzayani et al. 2019
Acid orange 7	Base-activated		34.53	97.9	Qi et al. 2016
Amaranth	CoFe_2_O_4_ NPs		35.8	100	Lin et al. 2018
Acid red 27 Acid yellow 17 Acid blue	CoTiO_3_	Oxone	52.1 53.8 82.8	100 ~ 100 80	Lin and Lin 2018

### Results of residual PMS and residual Fe after degradation

**Fig. 14:**
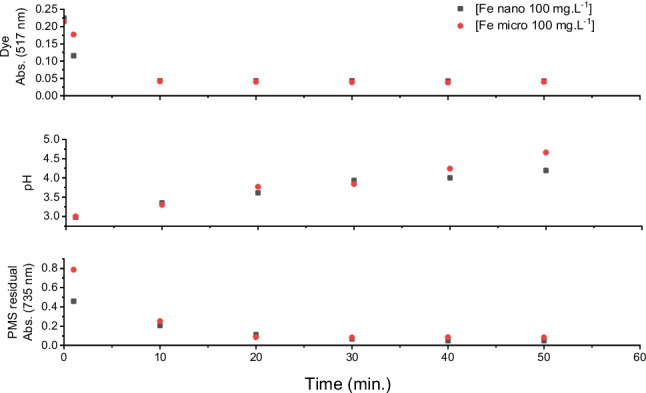
Degradation profile of RED 243 dye with 10 mg L^−1^ dye solution; 25 mg L^−1^ PMS; 100 mg L^−1^ micrometric and nanometric Fe^0^ at pH 3.0; and 50 mg L^−1^ PMS

For the PMS analysis, the standard conditions adopted were 10 mg L^−1^ dye concentration, 25 mg L^−1^ PMS, and 100 mg L^−1^ zero valent iron. After the addition of ABTS (10 mM) and Co^2+^ (10 mM) solutions, the solution turned dark green within a few seconds. As the degradation progressed, with each new aliquot removed, the dark green color gradually diminished, becoming increasingly translucent, reflecting the consumption of PMS in the system solution. The results are presented in Fig. 14.

A decay in PMS absorbance of approximately 90% was observed, reaching values close to 0.08 for micrometer-scale iron. This phenomenon may be associated with the generation of hydroxyl and sulfate radicals from the dissociation of PMS. As elemental iron oxidizes, these radicals are generated, and PMS becomes increasingly scarce. Thus, the solution’s colorimetric intensity becomes increasingly less greenish, indicating a decrease in PMS concentration over time, as expected.

Considering the final discharge at the end of degradation, for both types of elemental iron used (micrometric and nanometric), the final PMS absorbance values indicated a decay level above 85%. Using more dilute PMS solutions generated less sulfate residue in the solution at the end of the process.

**Fig. 15:**
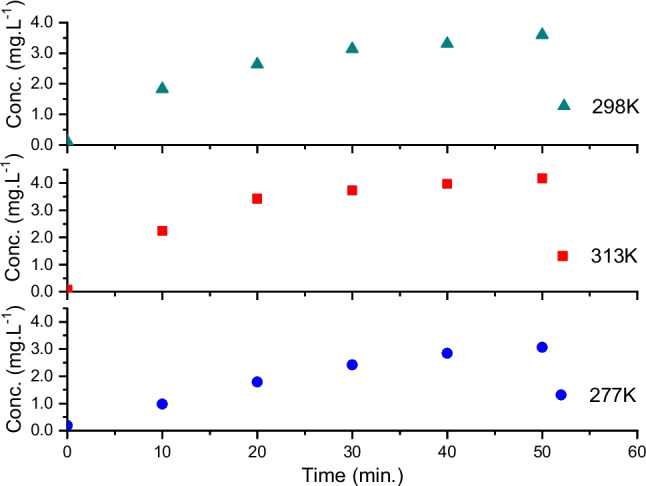
Residual iron concentration profile during degradation of RED 243 with 10 mg L^−1^ dye solution; 25 mg L^−1^ PMS solution; 100 mg L^−1^ micrometric Fe^0^

**Fig. 16:**
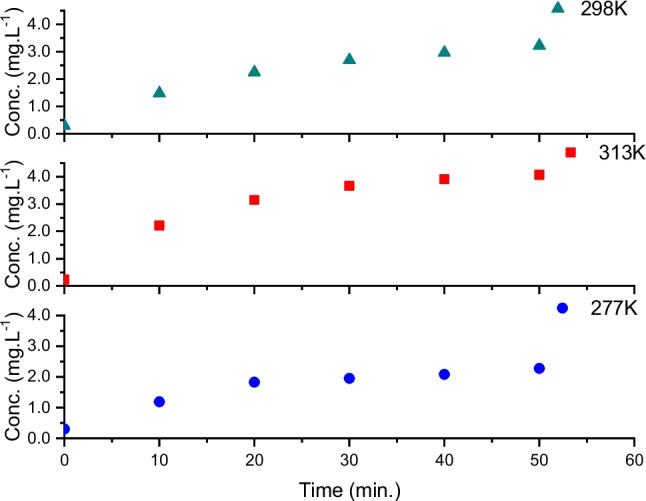
Residual iron concentration profile during degradation of RED 243 with 10 mg L^−1^ dye solution; 25 mg L^−1^ PMS solution; 100 mg L^−1^ nanometric Fe^0^

Regarding the residual iron analysis, it was observed that the iron concentration in the solution increases significantly over time, both at the micrometric and nanometric scales. This is due to the metal leaching into the most oxidized species in the solution, resulting in a more intense orange color over time. The residual iron concentration results are presented in Figs. 15 and 16.

At all temperatures analyzed, the residual iron concentration at the end of the 50-min test was higher when iron microparticles were used. This finding was consistent with the trend observed in this study, in which the use of iron micrometric particles results in greater interaction with the oxidizing agent, due to their purity, thereby favoring oxidation kinetics.

Furthermore, it was found that the presence of zero-valent iron particles favors catalysis of the dye-degradation reaction over time.

## Conclusions

It was found that the color degradation activity of the PMS solution is directly linked to the solution concentration and, consequently, to the volume of radicals generated. Degradation tests showed that the volume of PMS in the solution decreases, while the volume of iron increases over time.

Degradation efficiency, in turn, also depends on other reaction conditions, such as pH, wide temperature range, iron particle size, and others. The study found that higher concentrations of elemental iron and PMS result in faster and more efficient degradation. However, they also entail more severe reaction conditions.

The graphical behavior of dye degradation indicates that, relative to dye concentration, acetic acid increases the reaction rate more efficiently, a behavior linked to the smaller molecular size.

Kinetic studies indicated that the reaction system is pseudo-first-order, with a higher reaction rate and lower activation energy when using micrometric iron than when using nanometric iron, due to the purity of the reagents. It was also observed that the reaction rate increases with temperature, with the reaction with nanometric iron being more sensitive to this parameter.

Compared to the Fenton process using H_2_O_2_, PMS in an acidified system is faster and consumes less reagent, due to its higher oxidative potential and its predominance in the system.

Regarding the characteristics of the effluent generated after the test, it was observed that using micrometric iron in conjunction with the PMS allowed compliance with current legal standards for effluent disposal. Regarding the characteristics of the effluent generated after the test, at room temperature and without pH adjustment, we have a colorless solution, translucent in most cases, with both iron particles used.

## Data Availability

Data will be made available on request.
